# Overcoming Problems in the Practice of Public Health Among Tribals of India

**DOI:** 10.4103/0970-0218.58383

**Published:** 2009-10

**Authors:** Soudarssanane M Bala, D Thiruselvakumar

**Affiliations:** Department of Preventive and Social Medicine, JIPMER, Puducherry, India

## Introduction

India is home to almost half the tribal population of the world. Tribals are characterized by a distinctive culture, primitive traits, and socio-economic backwardness. The tribals of India, constituting 8.2% of the total population (84 million),(1) belong to around 698 communities or clans. Around 75 of these groups are called primitive tribal groups due to pre-agricultural level of knowledge, extreme backwardness, and a dwindling population.(2) However, the exact number of tribal groups may be lesser than 500 due to group-overlapping in more than one state.

Though the Indian tribals are a heterogeneous group, most of them remain at the lowest stratum of the society due to various factors like geographical and cultural isolation, low levels of literacy, primitive occupations, and extreme levels of poverty. Although scheduled tribes are accorded special status under the fifth/sixth schedules of the Indian Constitution, their status on the whole, especially their health still remains unsatisfactory. This paper explores the problems in delivering public health services to the tribal population of India and suggests solutions.

## Demography of the Indian Scheduled Tribes

Scheduled tribes are distributed throughout the country except Pondicherry, Haryana, Punjab, Chandigarh, and Delhi. Almost 25% of the Indian tribals live in Madhya Pradesh and Chattisgarh.(2)

The total population of scheduled tribes according to the 2001 Census is 84.3 million and has increased from 67.8 million in 1991, showing a decadal growth rate of 24.3%. The rate of growth has shown a declining trend (from 25.7% in 1981-1991) similar to that seen in the general population, but remains still higher than the national average of 21.3%. This declining trend in growth is seen in all tribal regions except the Southern states.(1)

The sex ratio of tribals is more favorable to females than the general population (972/1000 males vs. 927/1000). However, there is a wide variation among the different groups and states (1002 in Orissa to 889 in Goa).(1) The geriatric population (above 60 years of age) among tribals is 6.1%. Though this is actually an increase from 5.6% in 1981 in comparison to the general population (7.9%), the proportion is less. The dependency ratio among tribals is 83.9% and in the general population is 69%.(1) Literacy is increasing (47% in 2001 from 29.6% in 1991) but still lower than the general population (65%) and the gap between the literacy rates of STs and the general population continues almost at the same level of 17-18% for the last three decades. Almost 65% women are illiterate against the national figure of 46%.(1) High drop-out rates of 79% from formal education are a major problem.(3)

Around 91% of the tribal population still lives in rural area as against 72% for the whole nation.(1) The percentage of tribals living below poverty line is 47.3% in rural and 33.3% in urban areas, which is higher than the corresponding national figures of 28.3% and 25.7%, respectively.(3,4) The average tribal household size is 5.2 and is comparable to the national average of 5.3.(1) 81.6% of the total ST workers, both rural and urban, are engaged in the primary sector, essentially agriculture.

## Maternal and Child Health Indicators

There are vast differences in the health status of mothers and children between tribal and non-tribal populations. The indicators comparing the maternal and child health, highlighting the under-achievements among the tribals, are summarized in Table 1.(4,5)

**Table 1:** Maternal and child health indicators among tribals and others(4,5)

Indicator	Scheduled tribes		Others(5)
		
	NFHS 2	NFHS 3	NFHS 3
Median age at marriage	15.8 years	16.3 years	18.1 years
Awareness of legal age for marriage(4)	7.5%	-	22%(4)
Age at consummation of marriage	16.6 years	17.0 years	18.5 years
Total fertility rate	3.06	3.12	2.68
Median age at first childbirth	18.8 years	19.1 years	20.6 years
Proportion of pregnancies with no antenatal checkups	43.1%	37.8%	22.8%
Proportion of pregnancies with no TT immunization	25.8%	36.9%	16.4%
Proportion of pregnancies receiving IFA tablets	48.6%	62%	72%
Home deliveries	81.8%	82.3%	49%
Contraceptive use	39.1%	42.7%	51.4%
Proportion of births of order more than two	53%	51%	34.6%
Infant mortality rate/1000 LB	84.2	62.1	57
Neonatal mortality rate/1000 LB	53.3	39.9	39.1
Under 5 mortality rate/1000 LB	126.6	99.8	74
Exclusive breast feeding (median)	2.9 months	3.1 months	1.9 months
Completion of primary immunization	26%	31.3%	53.8%
No vaccination	-	11.5%	4.3%

Compared to the NFHS 2 survey,(4) the infant mortality, under-five mortality, and neonatal mortality have decreased,(5) the proportion of home deliveries is at a standstill. There was a fall in the median months of exclusive breastfeeding, while it had shown improvement among others from 1.3 months to 1.9 months.(4,5) The total fertility rate [Table 1] had shown a slight increase compared to the NFHS 2 survey.

## Other Public Health Problems Among Tribals

Malnutrition, as expected, is the most common health problem among tribals. In addition, communicable diseases such as tuberculosis, malaria, and STDs are major public health problems. Some tribal groups are also at high risk for sickle cell anemia. Generally tribal diets are seen to be deficient in protein, iron, iodine, and vitamins. A comparative analysis of the nutritional status of tribals and non-tribals is given in Table 2.(5)

**Table 2:** Nutritional parameters among tribals and nontribals(5)

Parameter	Scheduled tribes (%)	General population (%)
Malnutrition in children	54.5	33.7
Anemia in children	76.8	70
Anemia in women	68.5	51.3
Underweight among women	46.6	29.4
Vitamin A deficiency(6) women	30	18.5

According to the NFHS-3 survey,(5) 47% of tribal women are having chronic energy deficiency (CED) compared to 35% among the general population.

The most common diseases seen among tribals are respiratory tract infections and diarrheal disorders. 21% of children suffer at least two bouts of diarrhea every year and 22% suffer from at least two attacks of respiratory infections.(4) Tribals account for 25% of all malaria cases occurring in India and 15% of all falciparum cases.(6) Intestinal helminthiasis is widely prevalent among tribal children (up to 50% in Orissa and 75% in MP).(6,7) Skin infections such as tinea and scabies are seen among tribals due to poor personal hygiene.(6) Sexually transmitted diseases are relatively more common (7.2% prevalence of syphilis among Kolli hills tribals of Tamil Nadu).(8) The prevalence of tuberculosis is high, especially in Orissa.

Sickle cell trait prevalence varies from 0.5% to 45%, disease prevalence is around 10%. It is mostly seen among the tribals of central and southern India, not reported in North-East,(9,10) The prevalence of tobacco use is 44.9% among tribal men and 24% among tribal women.(11)

## Public Health Infrastructure

Although in tribal areas the government has provided for the establishment of primary health centers for every 20,000 population and sub-centers for every 3000 population, health care is not available to the majority of the tribals. This is due to several factors:(12)

Lack of accessibility to health facilities: As of the year 1999, 20,770 sub-centers, 3289 primary health centers, and 514 community health centers were available in the tribal areas. This leaves almost 25% of the tribal population without adequate access to health services even with the existing norms under the minimum need program.

Non-availability of health staff in the health centers: Almost 20% of the PHCs in tribal areas are not staffed with doctors (15% in non-tribal areas) and 15% of the posts of paramedical workers is vacant.

Quality of services: Non-availability of essential drugs and equipments, lack of proper building facilities, difficult terrain and constraints of distance and time (one ANM may typically be entrusted with 15-20 scattered villages), and lack of transport and communication facilities hinder the provision of health care.

Traditional practices and superstitions: Local beliefs, customs, and practices have obstructed health care delivery to the tribals. However, acceptance toward modern medicine is found to be increasing among tribals in the recent years.

## Solutions Suggested

The tribals of India are heterogeneous. Hence, the methods to tackle their health problems should not only be multi-fold, but also specific to the individual groups as feasible as possible. In this paper, several general approaches are described.

### Human resources development approach

One of the major problems in delivering health care to the tribals is shortage of manpower. Doctors and paramedical workers from the general population are reluctant to work in backward tribal areas. Further, there are not sufficient medical personnel hailing from the tribal communities, who will have a better understanding about the needs of their people and who may be more willing to work in such areas. Out of the available 26,509 doctors who pass out of medical colleges every year, there are not more than 1050, who belong to the ST group.(13,14) This is approximately 3.9%, whereas the proportion of tribals is 8.2% and this scenario has not changed in 2004.(3) Thus, there is a need to more than double the number of doctors qualified from among tribals (1875 admissions every year). Though there is a statutory provision of 7.5% reservation for tribals in medical education, apparently either the enforcement of this policy is not strictly done or there are not enough takers from the tribals for these seats. It is proposed that the proportion of distribution of these 1875 seats be worked out according to the proportion of the individual clans of tribals. The local clan communities may sponsor the requisite number of candidates for graduate medical education through the Ministry of Tribal Affairs at the state/central levels. In order to support this system, a parallel sponsorship at the middle and higher secondary school levels has to be actively undertaken, so that sufficient numbers of feeder-candidates are available. Though this is a long-term strategy, it will certainly help achieving the required results to a large extent - even granting that some proportion of such qualified doctors may tend to stay back in the developed urban areas.

Estimates on the public costs of providing graduate medical education are not available. A study done in Kerala during 1999 estimated that the total spending of the government for the whole MBBS course amounted to around Rs. 500,000. It is believed that the present graduate medical education may cost between Rupees 10 and 15 lakhs.(15) This entails a total cost of Rs. 1.8-2.8 billions (187-282 crores) for 1875 candidates from the ST community to the government. This expenditure is mainly borne by the Ministries of Health and Family Welfare at the central and state governments.

A similar ladder-like approach of sponsorship is recommended for post-graduate medical education, which should be need based particularly in the fields of tuberculosis and malaria (for example, one tuberculosis specialist, one senior tuberculosis laboratory supervisor, and one senior treatment supervisor per million tribals).

The situation is worse among other cadres of health workers. On the one hand, as such, the number of available paramedical education institutions is very less compared to the needs of the country. Only 13,000 ANMs are graduating every year.(16) A phenomenal increase is required in this area, which is the purview of general policy. Within this area, a parallel sponsorship cum educational opportunities has to be developed to cater for the needs of the tribal population.

The existing policies on reservation of medical seats do not meet the demands. If all the tribal areas in the country are to be covered by sub-centers and primary health centers with doctors and health staff from the tribal communities, a total of 4200 doctors, an equal number of pharmacists and laboratory technicians, 29,400 female health workers, 25,200 male health workers, and 4200 male and female health assistants are needed. If the primary health centers and sub-centers in the tribal areas are to cater for 5000 and 1000, respectively, (as the authors suggest in the paper later) a total of 16,800 doctors and 84,000 female health workers would be needed.

Though health is a state subject, the Centre has been given the authority of giving directions to the State Governments (Article 339(2), of the Fifth Schedule in the interest of the tribal population), which should be used to direct the State Governments to ensure provision of separate Tribal Sub Plans based on the percentage of tribals as recommended by the Ministry of Tribal Affairs.

### Empowerment approach

It is the authors' opinion that detailed quantitative and qualitative assessments of the agricultural and forest produce of the tribals are made. It is also suggested that the government should come forward with realistic procurement prices and arrange for timely procurement as well as redistribution of their agricultural/forest products. These measures will lead to not only a sustained and regular income, but also would encourage them to further their agricultural activities. The recent Tribal Cooperative Marketing and Development Federation of India Ltd. (TRIFED) is a welcome sign as provides marketing assistance and remunerative prices to STs for collection of minor forest produce (MFP) and surplus agricultural produce to protect them from exploitative private traders and middlemen. Irrigational and power support, along with modern communication and transport facilities are to be concentrated upon, on the lines of the Minimum Needs Program, and the special provision of funds under grant-in-aid under Article 275(1) of the Constitution has to be utilized for these developmental projects.

In those of the tribals where the system is matriarchal, it is envisaged to strengthen the system. However, among patriarchal tribals, measures like women's Self-Help Groups (particularly with economic yield) have to be introduced. A liberal financial policy of loans/grants must be evolved.

### Missionaries approach

Nutrition and education are basic accessories needed to modernize any community. A closely knit, Public Distribution System has to be developed, covering every interior pocket of the tribal areas, with a well-supported supply network. It is the authors' opinion that free distribution of both raw and fresh rations has to be implemented on a time frame, say for two generations; subsequently, this can be upgraded to the subsidized and later the fully paid strategy. The positive food fads of any given tribal community have to be addressed in this type of the public distribution system.

For example, a targeted PDS scheme similar to Anthyodaya Anna has to be devised, covering all tribal households. The food grain distribution has to be totally free and this must take care of their energy and protein needs. The present monthly per capita spending on food in the rural areas is Rs 299(17) (90% of the monthly per capita income), which has been shown to cover only 77% of energy needs. Hence, at least Rs.390 is to be spent in providing rations to each tribal individual. This would entail a total cost of Rs. 327 crores. The Village Grain bank Scheme provides funds for building storage facility, procurement of weights and measures and for the purchase of initial stock of one quintal of food grain of local variety for each family should be further strengthened by providing the rations as per the authors' recommendations given above.

On similar lines, schooling and education have to be developed fully utilizing the help of anthropologists and non-governmental organizations to inculcate the habit of universal education at the primary, middle, and higher secondary levels. Free distribution of educational and related items like books, uniforms, footwear, raincoats, sweaters, and bicycles along with supplementary nutrition has also been found successful in states like Tamil Nadu. The areas that need to be addressed are the inadequacy of the school buildings, both in number and in facilities, the lack of education in the mother language or dialect in primary classes, ignorance of non-tribals teachers about tribal languages and ethos, and the delay in the distribution of scholarships, textbooks, and uniforms.(3)

The authors consciously note that many methods suggested above are not entirely new ones and have been tried with success in vast sections of non-tribal areas. Hence, it is more of the administrative skills and organizational capabilities that need to be tuned up according to the tribal needs. This aspect is dealt within the section on administrative reforms.

### Administrative reforms approach

As noted in the earlier discussion, the setting of the official machinery is of prime importance. On the medical/health side, it is suggested that the population covered by the primary health centers and the sub-centers should be slashed down to 5000 and 1000, respectively, from the present norms of 20,000 and 3000. This is imperative given the difficult terrain and environment of the tribal topography. However, demanding this may appear to be in the initial stages, in the long run it would be possible to amalgamate these formations and upgrade them according to the needs of the times.

Similar administrative reforms in connection with Public Distribution System, School education, and establishment of women's Self help Groups have been highlighted earlier. The positive points from earlier research works in the demography and behavior of tribals - such as the favorable sex ratio, intercommunity marriages, non-consanguineous marriages, and positive acculturation behavior can be exploited and extended into other sections of tribals. For reasons of ensuring optimal attention for such developmental activities, the authors suggest the designation of the coming decade as the *Decade of Tribals* for appropriate raising, grouping, and redistribution of resources.
